# Phosphorylation of p23-1 cochaperone by protein kinase CK2 affects root development in *Arabidopsis*

**DOI:** 10.1038/s41598-019-46327-0

**Published:** 2019-07-08

**Authors:** Stefano D’Alessandro, Serena Golin, Sofia Zanin, Laura Cendron, Michela Zottini, Maria Ruzzene

**Affiliations:** 1 Department of Biology, University of Padova, Via U. Bassi 58/B, I-35131 Padova, Italy; 2 Department of Biomedical Sciences, University of Padova, Via U. Bassi 58/B, I-35131 Padova, Italy

**Keywords:** Plant sciences, Root apical meristem, Plant development, Kinases

## Abstract

Root growth is a fundamental process in plants and assures nutrient and water uptake required for efficient photosynthesis and metabolism. Postembryonic development of roots is controlled by the functionality of the meristem. Several hormones and signaling molecules regulate the size of the meristem, and among them, auxins play a major role. Protein kinase CK2, along with the chaperone protein HSP90, has been found to be involved in the regulation of auxin transport. Here, we show that p23-1, a cochaperone of HSP90, is phosphorylated by CK2 in Arabidopsis. We identified Ser201 as the major CK2 target site in p23-1 and demonstrated that phosphorylation of this site is necessary for normal root development. Moreover, we shed light on the nature of CK2 in Arabidopsis, showing that the three catalytic isoforms, CK2 *α*A, *α*B and *α*C, are proteins of approximately 40 kDa. Our results increase knowledge of the connection among HSP90, p23-1 and CK2 in Arabidopsis, suggesting the existence of a possible common root development mechanism controlled by these signaling molecules.

## Introduction

In addition to ensuring structural support to the aerial portion of the plant, roots provide nutrients and water^1^ and constitute environmental barriers between the plant and soil. Therefore, overall plant survival depends on appropriate root development, growth, and function^2^. Root growth is ensured by the perfect balance between cell division and differentiation at the level of the root meristem^3,4^. In turn, meristem maintenance is controlled by finely regulated orchestration of hormones and signaling molecules, with auxins acting as master regulators^1^.

The protein kinase CK2 is a Ser/Thr protein kinase that is usually organized as a tetrameric complex composed of two catalytic (α) and two regulatory (β) subunits^5,6^. In plants, the CK2 subunits are encoded by multigene families, with four genes encoding CK2α subunits (*αA*, *αB*, *αC*, also called *α1*, *α2* and *α3*, respectively, and *α4/cp*) and four genes encoding CK2β subunits in *Arabidopsis thaliana*^7,8^. In particular, CK2 αA, αB and αC have mainly nuclear localization both in roots and in leaves, whereas CK2 α4/cp localizes to plastids/chloroplasts^7,8^. The generation of an Arabidopsis *CK2 αAαBαC* triple knockout mutant, in which none of the nuclear subunits are expressed, resulted in late flowering, reduced hypocotyl growth, smaller cotyledon size, reduced number of lateral roots, and abscisic acid (ABA)-signaling defects^9^. A stronger impact on plant development was obtained by the generation of an inducible CK2 dominant-negative transgenic line, which was lethal upon long-term induction, confirming that CK2 activity is essential for plant growth^10^. Short-time induction, however, affected gravitropism, phototropism, lateral root formation and auxin transport due to the transcriptional misregulation of PIN-formed (PIN) and PINOID, resulting in lower auxin levels in the root meristem^11–13^. More recently, CK2 α4/cp single mutants have been shown to have stunted primary root growth and a shorter primary root meristem, where the CK2 α4/cp protein accumulates^8^.

The four Arabidopsis CK2β subunits (respectively β1, β2, β3 and β4), like the CK2α subunits, show a high degree of identity and localize to the nucleus and the cytosol, except CK2β4, which is nuclear-excluded.

CK2 phosphorylates more than 300 substrates with strict site specificity, requiring a precise amino acid sequence with an acidic residue at the n + 3 position downstream from the target Ser/Thr^14,15^. Among CK2 substrates, we have previously identified and characterized the p23-2 protein of Arabidopsis^16^. In Arabidopsis, two homologues of *p23* are present: *p23-1* (*At4g02450*) and *p23-2* (*At3g03773*)^17^. These two loci encode two proteins of different length, p23-1 (241 amino acids, 25.47 kDa) and p23-2 (150 amino acids, 17.4 kDa) respectively, which show a range of 38–60% similarity to other plant p23s^18^. The difference in length between p23-1 and p23-2 is due to a long glycine-rich segment in the C-terminal region of p23-1, the function of which is not yet understood. p23 is a cochaperone of HSP90^19^ and participates in multimolecular complexes with this protein along with many other cochaperones, such as HOP, AHA1, PP5, FKBPs (PPIases) and tetratricopeptide-repeat cochaperones, including SGT1b^17,20,21^. In animal systems, p23 is a small acidic protein that stabilizes the active conformation of the progesterone-receptor HSP90 complex by lowering the ATPase activity rate of HSP90^22–25^. In Arabidopsis, both p23 isoforms bind to HSP90. However, unlike their animal counterparts, they do not reduce the rate of ATPase activity of the chaperone^17^. We have previously characterized Arabidopsis p23s from a functional point of view^26^ and demonstrated that the absence of either p23-1 or p23-2 compromises auxin signaling and, ultimately, root development^26^. Specifically, we reported that in the double knockout mutant *p23-1 x p23-2* PIN transporters are mislocalized and expressed at a lower level compared with wild type (wt) plants. These results suggest a model of root growth regulation that involves HSP90 in controlling auxin transport through the interaction of both PIN and HSP90 with TWD1 (Twisted Dwarf 1)^21,27^. In addition, it was recently shown that a direct interaction exists between the auxin receptor TIR1 (Transport Inhibitor Response 1) and HSP90, strongly supporting a link between HSP90 regulation and auxin-controlled root growth^28^.

As mentioned above, through proteomic analysis, we identified Arabidopsis p23-2 as a CK2 substrate^16^. Here, we further investigate the p23/CK2 connection, showing that the long p23-1 isoform is phosphorylated by CK2 at higher levels than p23-2. In addition, we demonstrate that CK2-dependent phosphorylation of p23-1 at a specific phospho-site plays a key role in the regulation of root growth and development.

## Results

### p23-1 is phosphorylated at higher levels than p23-2 by a 40-kDa CK2-like kinase

The two homologues of p23 in Arabidopsis, despite showing a moderate level of similarity^17,18^, have many peculiarities that result in both isoforms being needed for correct root growth and development^26^. Both p23 isoforms contain CK2 phosphorylation consensus sites^16^, and we have previously demonstrated the phosphorylation of p23-2 by CK2^16^. Because p23-1 is the most abundant isoform and because both proteins play a role in root development^26^, we here investigated the possible phosphorylation of p23-1 by CK2.

**Figure 1:**
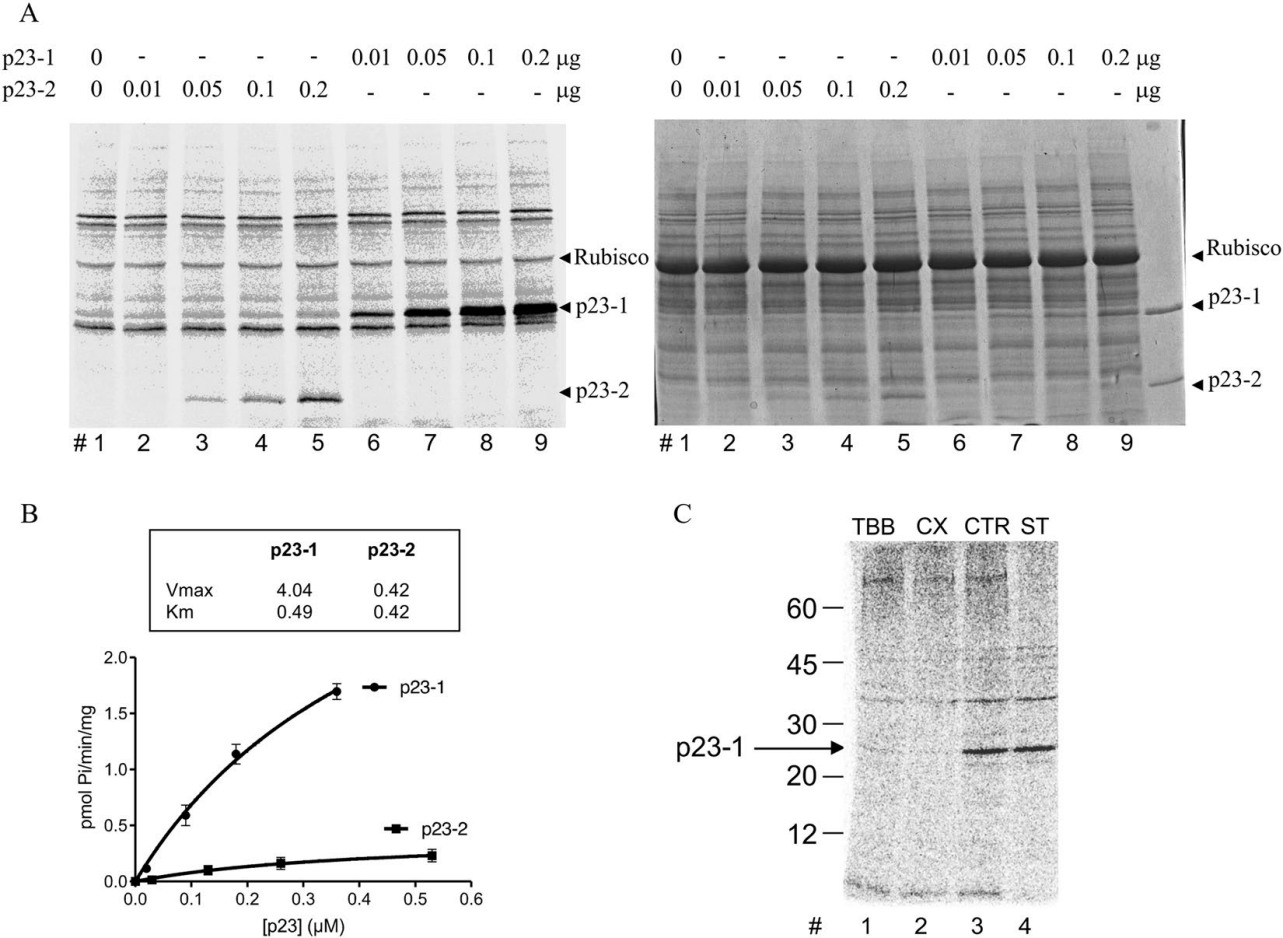
p23-1 phosphorylation by CK2-like activity. (**A**) Representative autoradiography (left) and Coomassie staining (right) after radioactive phosphorylation of increasing concentrations of recombinant p23-1 and p23-2, as indicated, by 10 μg of Arabidopsis total protein extract and separation via SDS-PAGE. The arrows indicate the migrations of each p23 isoform. Equally labeled radioactive bands in all lanes are due to autophosphorylation of the proteins present in the extract (see lane 1, where no p23 was present). The migration of the most abundant protein rubisco (~55 kDa) is also indicated. (**B**) Kinetics showing phosphorylation by CK2 with increasing concentrations of p23-1 and p23-2. The calculated kinetics values are shown in the box. Vmax is reported as pmol/min/mg, Km as μM. Quantification was performed by excising bands from the gel, as shown in panel A, and scintillator counting. Values are the means ± SD of independent experiments. (**C**) p23-1 (0.1 μg) was phosphorylated by 10 μg of Arabidopsis total protein extract in the presence (as indicated) of 2 μM TBB (TBB), 10 nM CX4945 (CX), or 1 μM Staurosporin (ST), or DMSO solvent as the control (CTR). Representative autoradiography is shown after protein separation by SDS-PAGE. The arrow indicates the migration of p23-1. Mw markers migrations are also shown on the left.

To this purpose, the coding sequence of the *At4g02450.1* locus was cloned into a prokaryotic expression vector to produce His-tagged p23-1 protein. We tested the *in vitro* phosphorylation of the recombinant p23-1 and compared it with the already characterized p23-2 in a radioactive phosphorylation assay, using Arabidopsis total protein extract as the source of kinases. By incubating increasing concentrations of the two recombinant proteins, we observed much more phosphorylation of p23-1, as highlighted by the autoradiography shown in Fig. 1A. The bands of interest, excised from the gel and analyzed in a scintillator counter, allowed us to draw the kinetic curves shown in Fig. 1B. The Km values (indicative of the enzyme-substrate affinity, representing the substrate concentration at which the reaction rate is half of Vmax) were very similar for the two isoforms (0.49 μM for p23-1 and 0.42 μM for p23-2), whereas the Vmax values differed by 10-fold (4.04 and 0.42 pmol min^−1^ mg^−1^, respectively, for p23-1 and p23-2; Fig. 1B).

To understand whether p23-1 was mainly phosphorylated by CK2, as was shown for p23-2^16^, we exploited the availability of specific CK2 inhibitors^29,30^ and the well-known insensitivity of CK2 to staurosporine, a compound that inhibits almost all other protein kinases^31^. We performed radioactive phosphorylation assays using recombinant p23-1 in the presence of the CK2 inhibitors TBB^32^, CX4945^29^, and staurosporine (respectively at 2 μM, 10 nM and 1 μM). As shown in Fig. 1C, the presence of staurosporine did not affect the phosphorylation of p23-1, whereas TBB or CX4945 almost completely abolished radioactivity incorporation in p23-1. These observations demonstrate that phosphorylation of p23-1 is largely dependent on the CK2-like activity present in the Arabidopsis total protein extract.

**Figure 2:**
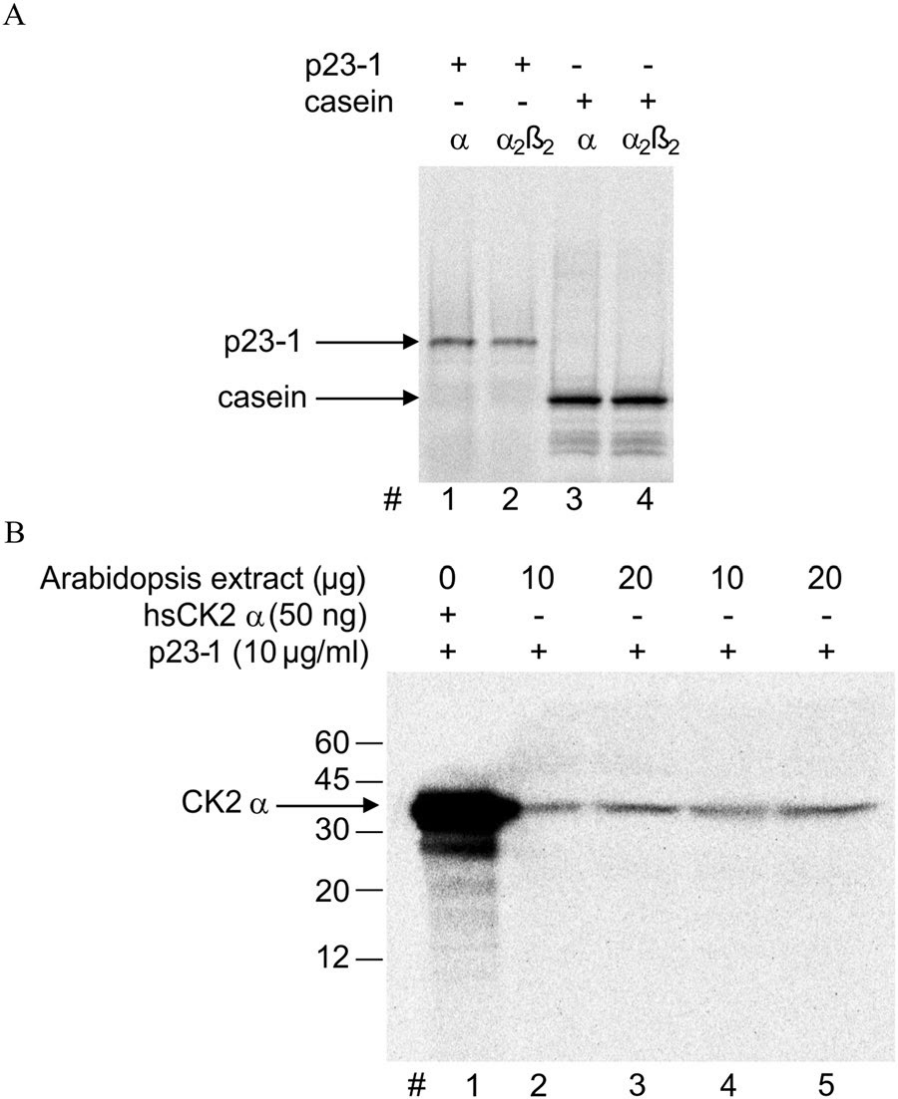
p23-1 phosphorylation by a monomeric 40-kDa CK2. (**A**) p23-1 (0.1 μg, lanes 1 and 2) was incubated with recombinant human monomeric CK2 (*α*, 15 ng) or tetrameric CK2 (*α*_2_β_2_, 5 ng); β-casein (1 μg, lanes 3 and 4) was used to ensure that the amount of each CK2 isoform chosen had the same catalytic activity towards a model substrate. After radioactive phosphorylation (10 min at 30 °C), samples were resolved by SDS-PAGE. A representative autoradiograph is shown. (**B**) Representative autoradiography of an *in-gel* kinase assay: 10 or 20 μg protein from Arabidopsis total extract (in duplicate, as indicated) was resolved by SDS-PAGE in which p23-1 (10 μg/ml) was included in the gel. In lane 1, human recombinant CK2 *α* (hsCK2*α* 50 ng, Mw 40 kDa) was loaded as a positive control. After electrophoresis and protein renaturation, the gel was incubated with a radioactive phosphorylation mixture and analyzed by autoradiography. The migration of Mw markers is shown on the left.

CK2 can act both as a monomer and as a tetramer composed of two catalytic and two regulatory subunits. *In vitro*, the tetrameric holoenzyme displays a higher activity compared to the isolated catalytic subunit on most substrates. Moreover, in animals, only a few proteins are phosphorylated by the monomer, whereas most physiological substrates are targeted by the tetramer^33^. However, the short p23 isoform, p23-2, is a better substrate for monomeric CK2^16^. To assess whether the same was true for the p23-1 isoform, we performed *in vitro* phosphorylation assays by exploiting the availability of recombinant monomeric and tetrameric human CK2, as the recombinant Arabidopsis CK2 isoforms were not available due to difficult purification^34^. In our experiments, we used monomeric *α* CK2 or tetrameric *α*_2_β_2_ CK2 in amounts corresponding to identical activity on β-casein (Fig. 2A, lanes 3 and 4). Under these conditions, we observed markedly more phosphorylation by *α* CK2 (Fig. 2A, compare lane 1 and 2). This result suggests that, at least *in vitro*, as for p23-2, the monomeric form of CK2 is the main form responsible for p23-1 phosphorylation. Next, we wanted to move to a more physiological context and identify the main p23-1 endogenous kinase in Arabidopsis. We performed *in gel* kinase assays, in which 10 μg/ml recombinant p23-1 was included in the gel and, after SDS-PAGE resolution of the Arabidopsis total protein extract and protein renaturation, phosphorylation of the substrate was performed by incubating the whole gel in a radioactive phosphorylation mixture, so that a labeled band could only appear where a p23-1-specific kinase had migrated.

As shown in Fig. 2B, the autoradiography of the gel shows only one radiolabeled band that migrated at approximately 40 kDa, comparable to the migration of the recombinant human CK2 *α* subunit, which was loaded as a positive control (Fig. 2B, lane 1).

### Analysis of the CK2 isoforms responsible for p23-1 phosphorylation and identification of the target site

**Figure 3:**
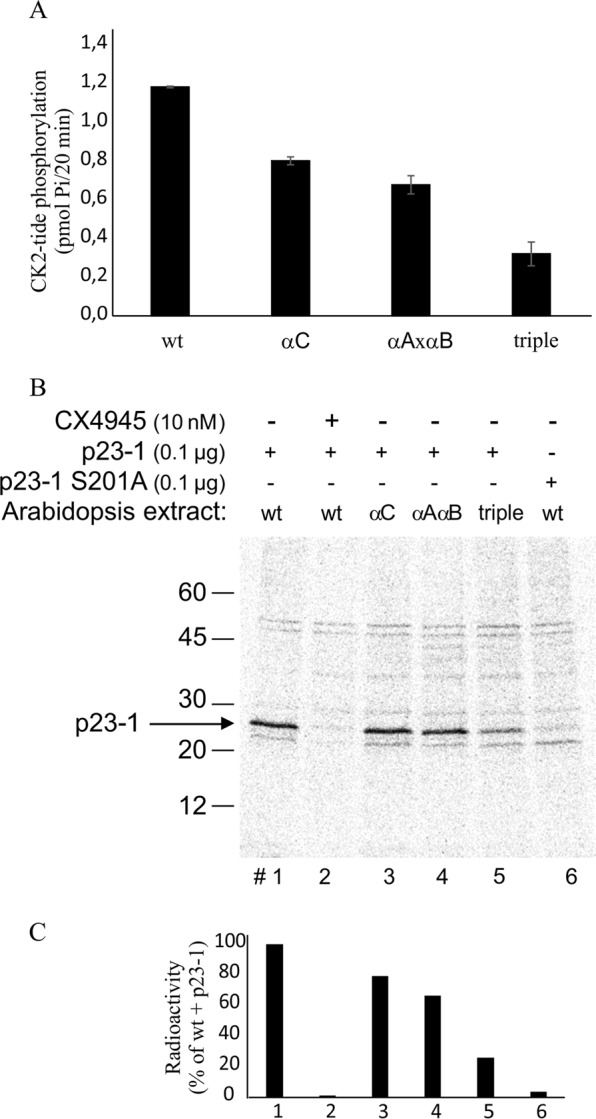
p23-1 phosphorylation activity of Arabidopsis mutant lines. (**A**) The specific CK2 peptide substrate CK2-tide was incubated with 10 μg total extract proteins from wild type (wt) or the *CK2 αC* mutant (*α*3), *CK2 αAαB* mutant (*α*A*α*B) or *of CK2 αAαBαC* (triple) mutant lines in the presence of a radioactive phosphorylation mixture. Blank controls were performed in the presence of 10 nM CX4945. Each phosphorylation activity is reported after subtraction of the relative blank control. (**B**) p23-1 wt or the S201A mutant (0.1 μg; lane #6) was incubated with 10 μg total extract proteins from wild type (wt), *CK2 αC* mutant (*α*C), *CK2 αAαB* mutant (*α*A*α*B) or the *CK2 αAαBαC* (triple) mutant, as indicated. CX4945 (10 nM) was present where indicated. A representative autoradiograph after protein separation by SDS-PAGE is shown. The migration of Mw markers is shown on the left. (**C**) Quantification of p23-1 radioactivity is shown, obtained by Cyclone Plus Storage Phosphor System (PerkinElmer) analysis from the gel of panel B.

The results presented in Fig. 2 strongly suggest that p23-1 is phosphorylated in Arabidopsis by a CK2-like monomeric kinase of approximately 40 kDa. There are four genes encoding CK2α subunits in *Arabidopsis thaliana*^7^. One of their products, CK2 *α*4/cp, localizes to plastids/chloroplasts, whereas the others, CK2 *α*A, CK2 *α*B and CK2 *α*C, are located in the nucleus. As p23-1 is detected both in the nucleus and in the cytoplasm^26^, plastidial/chloroplastic CK2 is not likely to be the physiological candidate kinase affecting p23-1 phosphorylation. To differentiate between the nuclear isoforms, we obtained homozygous Arabidopsis *CK2* mutants, impaired in the expression of CK2 *αA, αB or αC*, individually or in combination. As expected, these mutant lines showed reduced phosphorylation activity on the CK2 specific peptide CK2-tide^35^ (Fig. 3A). In particular, *CK2 αC* showed a 32% reduction in activity, whereas for *CK2 αAαB* and the triple mutant *CK2 αAαBαC*, the reduction was 43% and 73%, respectively. These results confirm that Arabidopsis *CK2* mutants are impaired in CK2 activity (some residual CK2-like activity was still present even in the triple mutant, possibly due to contaminating plastidial/chloroplastic CK2). We used total extracts of wt, *CK2 αC*, *CK2 αAαB* and *CK2 αAαBαC* as sources of endogenous kinases to perform an *in vitro* radioactive phosphorylation assay of the recombinant p23-1. As displayed in Fig. 3B,C (lanes 1, 3, 4 and 5), the phosphorylation level of p23-1 perfectly reflects the CK2 activity of the mutant lines, showing a decrease of 20% of the activity in the *CK2 αC* sample, of 35% in the *CK2 αAαB* sample, and of 74% in the triple mutant sample. These results, along with the complete inhibition of phosphorylation exerted by the CK2 inhibitor CX4945 (Fig. 3B, lane 2), further support the specificity of p23-1 phosphorylation by CK2 and demonstrate that all CK2 nuclear isoforms can catalyze p23-1 phosphorylation.

Analyzing the p23-1 sequence and considering the well-defined consensus for CK2 phosphorylation, we hypothesized that Ser201 is the major putative phosphorylation site. To evaluate this hypothesis, we produced a mutant protein with a phosphorylation-insensitive point mutation of Serine 201 to Alanine. The recombinant p23-1-S201A protein was then used as the substrate in a radioactive phosphorylation assay. As shown in Fig. 3B lane 6, the mutation completely abolished p23-1 phosphorylation. This result indicates that Ser201 is the major CK2 target site in p23-1.

### Expression profile of CK2 isoforms

**Figure 4:**
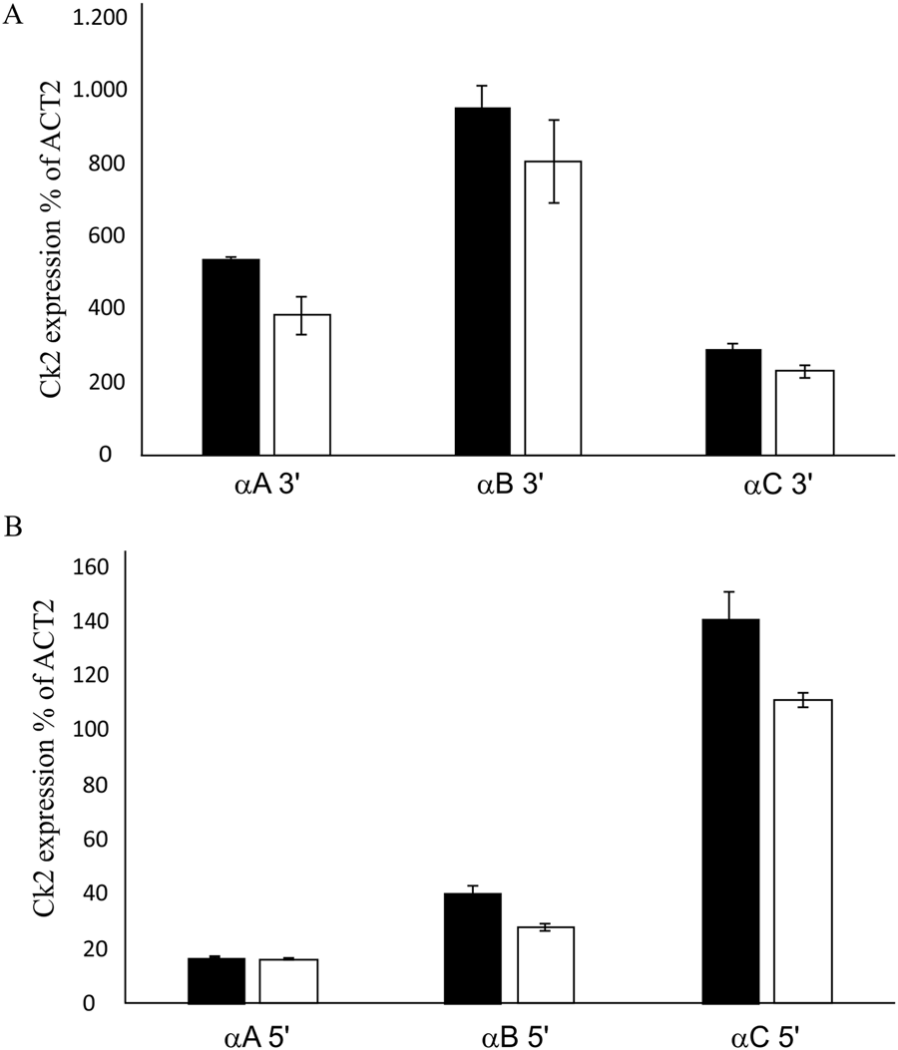
Arabidopsis CK2 nuclear catalytic isoform expression. (**A**) Expression levels of *CK2 αA*, *CK2 αB* or *CK2 αC* assayed by quantitative Real-Time PCR of the 3′-region of the mRNA. Values are reported as the percentage of the *Actin 2* (*ACT2*) expression level in the shoot (white bars) or root (black bars) of 10-day-old Arabidopsis seedlings. (**B**) Expression levels of *CK2 αA*, *CK2 αB* or *CK2 αC* assayed by quantitative Real-Time PCR on the 5′-region of the mRNA. Values are reported as the percentage of the *Actin 2* (*ACT2*) expression level in the shoot (white bars) or root (black bars) of 10-day-old Arabidopsis seedlings.

We demonstrated that p23-1 can be phosphorylated by all the three CK2 nuclear isoforms (Fig. 3), but *in vivo* phosphorylation of p23-1 can occur only if the kinase and substrate are expressed in the same tissue. We previously demonstrated that p23 isoforms are expressed in the root, where they mainly accumulate at the root meristem^26^. To understand which CK2 isoform is expressed in the root, we performed quantitative real time analysis on 10-day-old wt roots and shoots. Figure 4A shows the expression level of the three CK2 isoforms in the shoot (white bars) and in roots (black bars). Consistent with the results of Salinas *et al*.^7^, the nuclear isoforms of CK2 were ubiquitously expressed, suggesting that they are potentially able to phosphorylate p23-1 *in vivo*. The TAIR database (www.arabidopsis.org) predicts three long isoforms (CK2 *α*cp, *α*A and *α*B) with protein products of approximately 48 kDa and a short isoform, CK2 *α*C, of 38 kDa. However, by testing the expression levels of three *CK2* genes by qRT-PCR by analyzing the 5′- and 3′-regions of the mRNAs, we found strong differences. In fact, primers specific for the 5′-region of the mRNA, which encodes a longer N terminus of CK2 *α*A and *α*B, showed almost no expression (Fig. 4B) compared to the results of primers specific for the 3′-region (Fig. 4A).

### Phosphorylation of Ser201 is essential for p23-1 function

**Figure 5:**
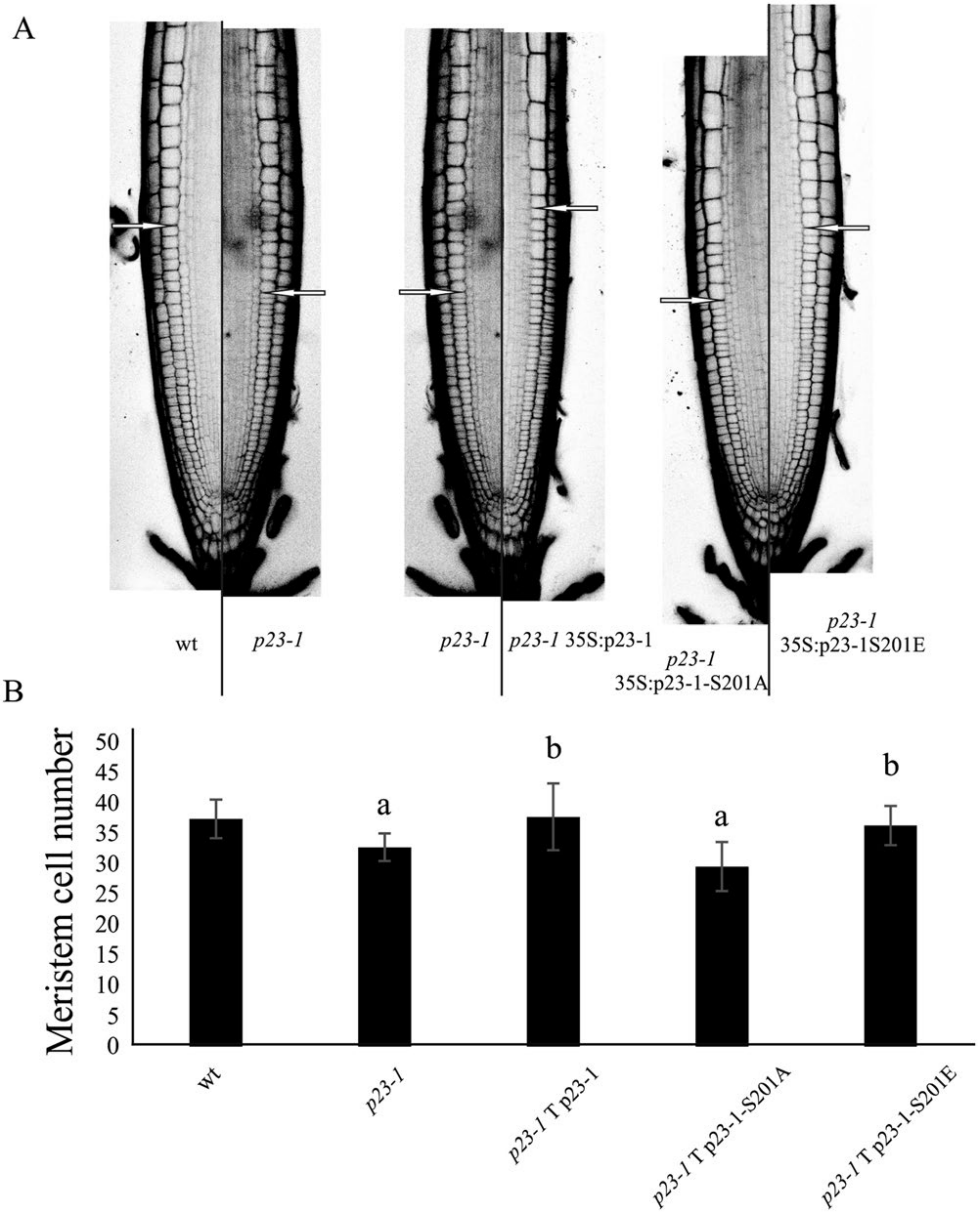
Impact of p23 phosphorylation on primary root development. (**A**) Propidium iodide stained roots of 10-day-old seedlings from the various lines: wild type (wt), single mutant (*p23-1*), complemented line (*p23-1* 35 S:p23-1), phospho-mimetic line (*p23-1* 35 S:p23-1-S201E), complemented mutant line (*p23-1* 35 S:p23-1-S201A). The excitation and emission wavelengths for confocal acquisition were 488 nm and 600–650 nm, respectively. The arrows show the upper limit of the meristematic zone. Scale bar = 50 μm. (**B**) The meristem cell number in the different genetic backgrounds. (^a^P < 0.01 compared with the wt, ^b^P < 0.01 compared with the background line; both Student’s t-test).

We previously demonstrated that p23 is involved in auxin-controlled root growth: we found that the reduced root length of the p23 mutant was reflected by a shorter meristem and a lower cell number^26^. The short meristem phenotype of the single *p23-1* mutant was rescued by overexpression of p23-1^26^. Based on these observations, we assessed whether the phosphorylation by CK2 of p23-1 is necessary for its function in root growth. We therefore exploited the p23-1 phospho-defective mutant S201A (Fig. 3) and also produced a putative phospho-mimetic mutant by replacing Ser201 with Glutamate (S201E). Both mutants were transformed into the *p23-1* mutant line, and their effects on the complementation of the root meristem were analyzed. As displayed in Fig. 5A,B, confocal analysis of propidium iodide-stained root meristem showed an average meristem length of 300 μm comprising 37 cortical cells in wt lines. The single mutant *p23-1* showed both a reduced meristem length (250 μm) and a lower number of meristematic cells (32), whereas the complemented line (*p23-1* 35 S:p23-1) showed no differences compared to the wt (300 μm and 38 cells). The phospho-mimetic line (*p23-1* 35 S:p23-1-S201E) complemented the mutant line (meristem size 285 μm and 36 cells), whereas the phospho-defective mutant line (*p23-1* 35 S:p23-1-S201A) did not, exhibiting a reduced meristem length and cell number (240 μm and 29 cells; Fig. 5A,B). These results strongly indicate that the phosphorylation of Ser201 is essential for p23-1 function in regulating the primary root meristem size and cell number.

## Discussion

Upon seed germination, the root apical meristem grows, as cell division prevails over differentiation. In Arabidopsis, approximately 5 days after germination, the final size is reached due to the balance between division and differentiation rates^4^. During this process, cross-talk occurs between cytokinin (promoting differentiation) and auxin (promoting division)^3,36–38^, in which p23-1 and -2 cochaperones are involved^26^.

Our study aimed at analyzing the role of p23 cochaperone phosphorylation on their function. We previously demonstrated that the short isoform p23-2 is phosphorylated by CK2 and that a physical interaction between this protein and the kinase can occur^16^. Here, we show that the long isoform p23-1 is phosphorylated by endogenous Arabidopsis kinases, for which it is a much better substrate than p23-2, with an almost 10-fold higher Vmax value (Fig. 1). Several observations indicate that the higher phosphorylation rate is mainly due to CK2 and not to the contribution of other kinases. First, the CK2-specific inhibitors TBB and CX4945^29,32^ completely prevent p23-1 phosphorylation by Arabidopsis total protein extract, whereas staurosporine, which is known to inhibit almost all protein kinases but not CK2^31^, is ineffective (Figs 1C, 3B). Second, in an *in-gel* kinase assay (Fig. 2B), we found that Arabidopsis total protein extract contains only a single band that is able to phosphorylate p23-1. This band migrates at approximately 40 kDa, similarly to the human CK2 catalytic subunit, which was loaded as a control. A third line of evidence that supports CK2 as a major p23-1 kinase in Arabidopsis comes from experiments with mutant plant lines. We compared wt Arabidopsis with lines in which the nuclear isoforms of CK2, *α*C, both CK2 *α*A and *α*B, or all CK2 *α*A, *α*B, *α*C isoforms were mutated. We found that the p23-1 phosphorylation activity of the total extracts from these lines reflected the activity towards a CK2 specific substrate, CK2-tide (Fig. 3). This result strongly suggests that phosphorylation of p23-1 is catalyzed by CK2 and highlights that all the isoforms are able to phosphorylate p23-1 *in vitro*. Moreover, consistent with the results of Salinas *et al*.^7^, we showed that all three nuclear catalytic subunits are expressed at a similar level in shoots and roots (Fig. 4A). Wang *et al*.^8^ reported that CK2 *α*4/cp is also expressed in roots, where it accumulates at the primary root meristem^7,8^. This result makes it plausible that the three nuclear CK2 *α* subunits interact with and phosphorylate p23-1, which also localizes in the nucleus and cytosol^26^.

Currently, we do not have an obvious explanation for the different Vmax values of p23-1 and p23-2 phosphorylation by CK2. However, this result is not unexpected since the affected sites are different^16^. We also speculate that, whereas p23-1 phosphorylation is catalyzed by all the CK2 catalytic isoforms, p23-2 might be more specifically targeted by a CK2 subset. Keeping in mind that root development requires both the p23-1 and p23-2 proteins^26^, it is worth noting that p23-1 is the most highly expressed isoform in Arabidopsis^26^. Therefore, despite their similar Km values (thus presumably similar phosphorylation at low concentrations), the higher Vmax of p23-1 would ensure more efficient phosphorylation at higher concentrations. Because the effect of any phosphorylation obviously depends on its extent, this is expected to be relevant in controlling the physiological function of p23-1. Furthermore, the regulatory potential is further supported by the observation that the phospho-site is located in the C-terminal region, which is only present in p23-1 and has been reported to be a regulatory module in the case of human p23^39^.

The TAIR database predicts three long isoforms of CK2 of approximately 48 kDa and a short isoform of 38 kDa. However, we found that the 8-kDa-longer N termini of CK2 *α*A and *α*B are expressed at low levels compared to the 3′-terminus of the mRNA. This finding is consistent with the results of Salinas *et al*.^7^, who also described Arabidopsis CK2 as 40 kDa. Furthermore, considering that the three enzymes are active on p23-1 (see mutant lines, Fig. 3B,C) and that the *in-gel* kinase assay shows a single 40-kDa band phosphorylating p23-1 (Fig. 2B), we conclude that the three nuclear CK2 forms are 40-kDa and not 48 kDa-sized proteins, which implies that, under our experimental conditions, we can exclude multiple kinases targeting p23-1, but we cannot discriminate between different CK2 isoforms.

A final confirmation of the contribution of CK2 on p23-1 phosphorylation is the identification of the target site Ser201 (Fig. 3B), which perfectly fits the CK2 consensus sequence and, when mutated, completely prevents phosphorylation.

Identification of the phosphorylated site prompted us to investigate whether it was involved in the modulation of the function of p23-1. We previously demonstrated that p23-1 is fundamental for root meristem growth since mutation of this gene causes defective root development. Here, we exploited a phospho-defective mutant, *p23-1* 35 S:p23-1 S201A, and a phospho-mimetic mutant, *p23-1* 35 S:p23-1 S201E, to understand the role of p23-1 phosphorylation in this process. We found that the phospho-mimetic protein p23-1 S201E was able to complement the mutant and that the phospho-defective protein p23-1 S201A showed no rescue of the root meristem phenotypes. Therefore, we conclude that p23-1 phosphorylation at Ser201 by CK2 is essential for normal root development in Arabidopsis.

p23 cochaperones are known to function either by themselves or by mediating the action of HSP90 in multimolecular complexes^40^. The human homolog of p23, formerly known as cPGES (cytosolic prostaglandin E synthase), stabilizes the HSP90 complex and displays autonomous enzymatic activity. Interestingly, human p23 has also been reported to be a substrate of CK2, which phosphorylates Ser113 and Ser118, leading to increased prostaglandin E3 ligase activity^40,41^. For Arabidopsis p23-1, future studies will be necessary to understand the exact mechanism of regulation by CK2.

It is already known that CK2 defective Arabidopsis mutants display late flowering, reduced hypocotyl growth, a smaller cotyledon size, reduced number of lateral roots, and ABA-signaling defects^9^. Moreover, short-term induction of a CK2 dominant-negative mutant results in auxin deficient phenotypes in the root, as reported for the *CK2 α4/cp* mutant line^8^. Previous work anticipated a close linkage between CK2 and salicylic acid (SA) signaling in Arabidopsis^42^, and a more recent work^13^ shows that CK2 mediates the cross-talk between auxin and SA signaling. Here, we add further complexity to the CK2/auxin connection by demonstrating that p23-1 phosphorylation by CK2 is also involved. p23-1 and p23-2 cochaperones are part of the HSP90 complex, possibly participating in auxin sensing (by stabilizing the auxin receptor TIR1^28^), and in auxin transport (by interacting with TWD1^27^). Both of these possibilities are compatible with the decreased expression of PIN1 and PIN7 found in the p23 mutants^26^. We therefore propose a model in which CK2 is necessary for normal root growth and development, not only to reduce SA biosynthesis but also to actively phosphorylate p23-1.

## Material and Methods

### Chemicals

The CK2 inhibitor CX4945^29^ (5-[(3-Chlorophenyl)amino]-benzo[c]-2,6-naphthyridine-8-carboxylic) was purchased from Glixx Laboratories.

Staurosporine was purchased from Sigma-Aldrich. Recombinant human and maize CK2 proteins were produced, purified and kindly donated by Stefania Sarno^43^. Purified proteins were dialyzed against 25 mM Tris pH 7.5 and 50% glycerol and stored at −20 °C. Radioactive ATP came from PerkinElmer.

### Plant material and growth conditions

All experiments were performed on *Arabidopsis thaliana* ecotype Columbia (Col-0). The mutants *CK2αA* (SALK_104075), *CK2αB* (SALK_129331), CK2*αC* (SALK_151200), *CK2αAαB* and *CK2 αAαBαC* were kindly provided by Prof. Enamul Huq^9^. The mutant *p23-1.1*(SAIL 245_H06) was obtained from the European Arabidopsis Stock Centre (NASC)^26^. Seeds were surface sterilized in 70% ethanol plus 0.05% Triton X 100 and then in 100% ethanol. Seeds were plated in square petri dishes containing Murashige and Skoog medium (MS^−^½)^26^ supplemented with 0.5 g/l MES-KOH pH 5.7, 0.8% Plant Agar (Duchefa), and 1% sucrose; stratified for 2 days at 4 °C in the dark, and placed to grow vertically in a growth chamber under a long-day light period (16 h light/ 8 h dark) at 150 μmol m^−2^ s^−1^. Complemented lines *p23-1.1* 35 S:p23-1, *p23-1.1* 35 S:p23-1-S201A and *p23-1.1* 35 S:p23-1-S201E were generated by transforming the single *p23-1.1* mutants with Agrobacterium harboring the coding sequence (CDS) of *p23-1* or the phospho-defective or phospho-mimetic point mutation constructs. The primers for CDS cloning and mutation are reported in Supplementary Table 1. Point mutagenesis was performed as described in Edelheit *et al*.^44^.

### RNA isolation and qRT-PCR

Seven days after germination, roots and shoots of wt and mutant seedlings were separately harvested for subsequent analyses. Total RNA extraction from plant samples was performed using TRIzol® Reagent (Invitrogen) following the manufacturer’s instructions. First-strand cDNA synthesis was performed using 1 μg of RNA, oligo(dT) primers and SuperScript-II Reverse Transcriptase (Invitrogen) following the manufacturer’s instructions. The primers for qRT-PCR (quantitative Real Time PCR) are reported in Supplementary Table 1.

### Production of recombinant proteins

To purify the recombinant protein p23-1, p23-1-S201A and p23-2, the coding sequences of *At4g02450.1* and *At3g03773.1* were PCR-amplified using Phusion DNA polymerase and cloned in the pET28 vector downstream of the 6xHIS tag. The vectors pET28-p23-1, pET28-p23-2 and pET28-p23-1-S201A were transformed into BL21(DE3) *E. coli* cells for expression. The 6His-tagged proteins were expressed and purified by nickel affinity chromatography (HIS-Select® Nickel Affinity Gel, from Sigma-Aldrich) starting with 1 L cultures. The affinity-purified proteins were subjected to gel filtration (Superdex 200 HR 10/30 or HR 16/60 column, GE Healthcare, equilibrated in 30 mM Tris-HCl, pH 8, 150 mM NaCl elution buffer). For each purification, the eluted fractions were pooled and concentrated by centrifugal filters (Vivaspin® Centrifugal Concentrators, 10,000 MWCO, from Sartorius Stedim Biotech), yielding a final concentration ranging from 100 μg/ml to 4 mg/ml. Purified proteins were analyzed by 12% SDS-PAGE and Bradford protein quantification.

### *In vitro* phosphorylation assays

Recombinant protein substrates were incubated for 20 min at 30 °C in the presence of 10 μg of the total protein extract from seven-day old Arabidopsis seedlings or recombinant monomeric (*α*) or tetrameric (*α*_2_β_2_) human CK2 in a phosphorylation buffer composed of 50 mM Tris–HCl, pH 7.5, 10 mM MgCl_2_, 40 μM ATP, and [γ-^33^P] ATP (1000–2000 cpm/pmol). NaCl (100 mM) was added when tetrameric CK2 was used. Further details are provided in the figure legends. After incubation, samples were loaded onto SDS-PAGE gels, which were stained with Coomassie blue and analyzed by autoradiography with the Cyclone Plus Storage Phosphor System (PerkinElmer). When quantification of the total protein extract on the CK2 specific peptide CK2-tide (RRRSDDSDDDDD) was required, the whole reaction mixture was transferred onto phospho-cellulose paper and counted in a scintillation counter^35^. Kinetic values were calculated with the GraphPad Prism Software.

### *In gel* kinase assay

For this assay, 10 μg/ml p23 was included in a 15% SDS-PAGE gel in which Arabidopsis total protein extracts (40 μg) were separated^45^. After electrophoresis, SDS was removed, and proteins were renatured^16^. Then, the gel was incubated in phosphorylation buffer composed of 50 mM Tris–HCl, pH 7.5, 10 mM MgCl_2_, 20 μM ATP, and [γ-^33^P]-ATP (specific radioactivity 1000–5000 cpm/pmol). After Coomassie blue staining, the gel was analyzed by autoradiography for the detection of radioactive bands.

### Confocal microscopy

Seven-day-old seedlings were mounted in 2% (20 μg/ml) propidium iodide (PI; Sigma Aldrich) solution on a microscope slide, and images were acquired on a LEICA SP5 laser scanning confocal microscope. The excitation and emission wavelengths are reported in the figure captions. High definition images were acquired (1024 × 1024, 25X water-immersion objective) and analyzed by the Fiji – ImageJ software. Experiments were performed at least in triplicate, and each sample set consisted of 10 seedlings.

### Statistics

All experiments were performed at least in triplicate, and pictures represent typical examples. Values are provided as the mean ± SD. Statistical significance was inferred from Student’s t-test.

## Supplementary information

Supplementary Information

## Data Availability

The Arabidopsis lines and all the material generated in this study are available from the corresponding author (MZ: michela.zottini@unipd.it), upon reasonable request.
