# Supplementary material for: Phosphorylation of p23-1 cochaperone by protein kinase CK2 affects root development in Arabidopsis
Source: Sci Rep. 2019 Jul 8;9:9846. doi: 10.1038/s41598-019-46327-0 (PMC6614504; doi:10.1038/s41598-019-46327-0)
Supplement: Supplementary file 1 — Supplementary Information [file 41598_2019_46327_MOESM1_ESM.pdf]

Phosphorylation of p23-1 co-chaperone by protein kinase CK2 affects root development in *Arabidopsis*

Stefano D'Alessandro, Serena Golin, Sofia Zanin, Laura Cendron, Michela Zottini and Maria Ruzzene

Supplementary information

Quantitative Real Time PCR

HSP90.2	ACTCACCGTGCTGTCTTGTA
HSP90.2	GAGGAGAAGCACAAGGTCCT
CK2A1	GTTACTGTGCGCCGTCTTAG
CK2A1	AACCGGAAACTTCCGAGACA
CK2A2	TCTTCGACGGTACCTGTTGTT
CK2A2	TCAACGGTTCCGGTGTTGAC
CK2A3	TCTCGTTGTTCAATGGGGTCA
CK2A3	ACGCAACGCTCATTCGTATTG
CK2A1	TGATCCACAACTGGAAGCACT
CK2A1	ATCAATCGCCTCGGGTGAAA
CK2A2	TGACAACCAGGATCAGCTCG
CK2A2	AGGCTTCCTGCTATGTCTCC
CK2A3	GGTCTCACCTGAGGCAATTGAT
CK2A3	TGCTCTCTGCGGCTTTAACT
ACT2	CATTCCAGCAGATGTGGATCTC
ACT2	ACCCCAGCTTTTTAAGCCTTTG

Cloning

At3g03773	CATGCATATGAGTCGTAATCCGGAGGTTCTT
At3g03773	CATGCATATGCTACTTGTTTCTTGCCTTTTCCA
At4g02450	CATGCATATGATGAGTCGTCATCCTGA
At4g02450	CATGCTCGAGTCACTTGTCTTCCTTAAC

Point mutagenesis

At4g02450	GGAAGAGTTTGAAGACGCTGATGATGAAGAAG	point mutation S ->A
At4g02450	GGAAGAGTTTGAAGACGAGGATGATGAAGAAG	point mutation A -> E or S -> E
